# Supplementary material for: Emergency medical service interventions and experiences during pandemics: A scoping review
Source: PLoS One. 2024 Aug 1;19(8):e0304672. doi: 10.1371/journal.pone.0304672 (PMC11293743; doi:10.1371/journal.pone.0304672)
Supplement: S2 File — (DOCX) [file pone.0304672.s002.docx]

**S2 file:** **Ambulance (Emergency Medical Service) interventions in response to pandemics: a scoping review protocol**

*Ffion Curtis, Withanage Iresha Udayangani Jayawickrama, Despina Laparidou, Dedunu Weligamage, Rob Spaight, Emma Horncastle, Marishona Ortega, Aloysius Niroshan Siriwardena*

Scoping reviews can be published/registered on either of these:

<https://osf.io/>

<https://figshare.com/>

# **Background**

Infectious disease pandemics spread rapidly across the globe resulting in widespread disease and significant burden on healthcare systems . The infection due to the SARS-CoV-2 virus strain was first identified in Wuhan China in December 2019, and on March the 11^th^ the World Health Organization (WHO) declared the outbreak of this coronavirus disease, COVID-19, to be a global pandemic. The global impact of COVID-19 has been profound, and it represents the most significant public health threat seen in a respiratory virus since the H1N1 influenza pandemic in 1918 (Imperial College London COVID-19 Response Team). Whilst our understanding of infectious diseases has advanced since the H1N1 1918 pandemic, many of the challenges we now face are similar. Lessons learnt from previous pandemics, such as the Severe Acute Respiratory Syndrome (SARS-CoV) 2002, Middle East Respiratory Syndrome (MERS) 2012 and H1N1 2009 pandemics, are essential for informing global pandemic preparedness plans (WHO, 2005). Pandemic preparedness is a crucial issue for public health worldwide.

Emergency Medical Services (EMS) have an integral role within the emergency preparedness system. A recent study reported that EMS staff felt that there was a lack of training in relation to future pandemics (Rebmann et al., 2019), and several factors were identified (such as perceptions of an infectious pandemic, and feeling safe working during pandemic) that would influence their willingness to work during a pandemic. An evaluation of pandemic preparedness training for EMS (to include an educational intervention focused on routes of virus transmission, reviewing policies regarding infection control practices, and proper use of respiratory personal protective equipment [PPE]) reported an increase in knowledge among staff, but concluded little was known with regards to how this would translate into behaviour modification (Gershon et al., 2009). EMS are on the front line to respond to the urgent medical needs of patients throughout an influenza pandemic. Despite this, there appears to be sparse evidence in this area.

The aim of this scoping review is to identify and present the available quantitative and qualitative evidence of EMS pandemic preparedness, and how this translates into practice. This will include studies of EMS pandemic preparedness plans, intervention implementation, evaluations, and importantly perceptions of EMS staff and patients. The findings of this scoping review will be used to inform future research to strengthen EMS pandemic preparedness planning.

The JBI scoping review guidelines, and the Arksey and O’Malley methodology framework have informed the development of this scoping review protocol, which was written in accordance with the PRISMA extension for scoping reviews (PRISMA-ScR) checklist.

# **Research questions**

What interventions (e.g. infection control, PPE) are implemented within the Emergency Medical Services (EMS) in response to/during pandemics?

What outcomes are reported relating to EMS interventions in response to pandemics?

What qualitative evidence is there describing the experiences of EMS staff and patients during pandemics?

# **Identification of relevant studies**

The search strategy will be informed by the inclusion criteria. These may be adapted based on initial searches:

Inclusion criteria for qualitative studies:

- Participants: Ambulance service/EMS staff; patients; relatives.
- Concept (phenomena of interest): This review will consider studies that discuss the experiences of EMS staff and/or pre-hospital patients during epidemics or pandemics.
- Context: All international prehospital EMS/ambulance services.
- Types of study: Qualitative designs including, but not limited to, phenomenology, grounded theory, ethnography and a generic qualitative approach.

Inclusion criteria for quantitative studies:

- Participants: Ambulance service/EMS staff; patients, attended by ambulance service/EMS staff during epidemics or pandemics.
- Concept: This scoping review will consider studies that include any type of intervention implemented in response to epidemics or pandemics within prehospital EMS/ambulance services.
- Context. All international pre-hospital emergency medical services/ambulance services.
- Types of study: Quantitative approaches including, but not limited to, interventional studies, observational studies (cohort and case control), cross sectional studies and surveys.

Multi methods studies:

- Must meet qualitative and/or quantitative inclusion criteria as above. Multi-methods studies included will have their qualitative and/or quantitative data extracted into their respective arms

of this scoping review.

Key search terms will include: ambulance; emergency medical services; and pandemic. The following databases will be searched from inception: MEDLINE, PubMed, CINAHL, Cochrane Library, PsycINFO and PROSPERO. Database searches will be supplemented with internet searches (i.e. Google Scholar), and forward and backward citation tracking from included studies and review articles. Studies will be included if they report quantitative and/or qualitative data, and are published in English.

# **Study Selection**

A two-part study selection process will be used: (1) a title and abstract review and (2) full-text review. Titles and abstracts will be independently screened by two reviewers in line with inclusion criteria. Full text papers will be retrieved and assessed by two reviewers when studies are not excluded based on title and abstract. Any discrepancies will be resolved with the inclusion of a third reviewer.

# **Charting the data**

The fourth stage of Arksey and O’Malley scoping review methodology is the charting of the data of the selected articles. This will enable us to identify, characterize, and summarize the evidence, including identification of research gaps. A standardized, pre-piloted form will be used to extract data from the included studies. One reviewer will undertake data extraction for each study, with a second reviewer cross checking all extracted data.

During this stage, key information about the selected articles will be collected to include: study details (title, author, date), methods (aims, objectives, research questions, study design, setting, data collection methods, intervention characteristics, outcomes, data analysis, context in terms of findings and/or relevant theory), and participants (demographics, inclusion/exclusion criteria, method of recruitment, sample selection and sample size).

Quality assessment: whilst not an essential component of a scoping review, this process will add value as it may provide recommendations for future researchers with regards to the design, conduct and reporting of research in this critically important area.

# **Collating, summarizing and reporting the results.**

As a scoping review, the purpose of this study is to aggregate the findings and present an overview of the research. Where appropriate a descriptive qualitative content analysis to include basic coding of data will be conducted to analytically map participant perceptions of ambulance service interventions. The results will be presented in tabulated and/or charted format. The evidence will be classified under main conceptual categories, such as: “intervention trigger”, “intervention type”, “aims”, “methodology adopted”, “key findings” (evidence established), and “gaps in the research”, accompanied by a descriptive narrative summary.

# Contact details for further information

Ffion Curtis [fcurtis@lincoln.ac.uk](mailto:fcurtis@lincoln.ac.uk)

# Organisational affiliation of the review

University of Lincoln

# Review team members and their organizational affiliations

Dr Ffion Curtis. University of Lincoln

Dr Withanage Iresha Udayangani Jayawickrama. University of Lincoln

Ms Despina Laparidou. University of Lincoln

Dr Dedunu Weligamage. University of Lincoln

Rob Spaight, East Midlands Ambulance Service NHS Trust

Emma Horncastle, East Midlands Ambulance Service NHS Trust

Miss Marishona Ortega. University of Lincoln

Professor Aloysius Niroshan Siriwardena. University of Lincoln

# Type and method of review

Synthesis of quantitative and qualitative studies, Systematic review

# Anticipated or actual start date

1 May 2020

# references

Barnett et al., (2010) Gauging U.S. Emergency Medical Services Workers' Willingness to Respond to Pandemic Influenza Using a Threat- and Efficacy-Based Assessment Framework. PLOS ONE <https://doi.org/10.1371/journal.pone.0009856>

Gershon et al., (2009) Evaluation of a pandemic preparedness training intervention of emergency medical services personnel. Prehosp Disaster Med. 24(6):508-11.

Impact of non-pharmaceutical interventions (NPIs) to reduce COVID19 mortality and healthcare demand. Imperial College London COVID-19 Response Team. 2020. Accessed online 05/04/20 <https://www.imperial.ac.uk/media/imperial-college/medicine/sph/ide/gida-fellowships/Imperial-College-COVID19-NPI-modelling-16-03-2020.pdf>

Rebmann et al., (2019) Emergency Medical Services Personnel’s Pandemic Influenza Training Received and Willingness to Work during a Future Pandemic. Prehospital Emergency Care. <https://doi.org/10.1080/10903127.2019.1701158>

WHO global influenza preparedness plan: The role of WHO and recommendations for national measures before and during pandemics. 2005. Accessed online 05/04/20 <https://www.who.int/csr/resources/publications/influenza/WHO_CDS_CSR_GIP_2005_5.pdf>
