# Supplementary material for: Emergency medical service interventions and experiences during pandemics: A scoping review
Source: PLoS One. 2024 Aug 1;19(8):e0304672. doi: 10.1371/journal.pone.0304672 (PMC11293743; doi:10.1371/journal.pone.0304672)
Supplement: Attachment — Submitted filename: Review of PLOS ONE PONE-D-23-22267.pdf [file pone.0304672.s003.pdf]

Summary of the research

This is an excellent scoping review of EMS interventions and experiences in a pandemic / epidemic / outbreak context with some moderate need for revision. The aim of the authors was to "identify and present the available … evidence of EMS pandemic preparedness, and how this translates into practice." They hope the findings will inform future research and strengthen policy/practice. The authors searched 6 databases and created a narrative synthesis of all eligible quantitative, qualitative and mixed methods studies. Results were presented in terms of main themes resulting from this review for quantitative and qualitative "arms" of review seperately. 22599 citations were whittled down to 90 pertinent studies.

Quantitative: 7 themes (abstract)

Qualitative: 5 themes (abstract)

Main findings were concern for self and family safety expressed by EMS workers. Although there were concerns for knowledge and training as well and appropriate PPE, EMS personnel were generally "willing and prepared" to report to duty.

This research fills an important gap in the literature, however one narrative review (using same basic framework of PRISMA-Scr, however much more limited in scope and focused on EMS calls only) is out there:

Alaa Al Amiry & Brian J Maguire (2021) Emergency Medical Services (EMS) Calls During COVID-19: Early Lessons Learned for Systems Planning (A Narrative Review), Open Access Emergency Medicine, 13:, 407-414, DOI: 10.2147/OAEM.S324568

Although not critical, consider citing this study in introduction along with [5] and [6].

1. Based on publication criteria this manuscript will meet all criteria with several minor to moderate edits and clarifications.
2. Meets use of PRISMA-Scr checklist as required for best practice in scoping reviews. Unclear from the manuscript why Arksey and O'Malley was also used although the framework provided therein is rich, detailed and I'm sure was of use to the authors as it is somewhat focused on "the fourth type of scoping study, aimed at identifying gaps in the existing research literature." Per my reading this manuscript is a blend of both the 3rd and 4th types of scoping reviews as detailed by Arksey and O'Malley.
3. Appreciate reflexivity statement, but unclear as to how (for this type of publication specifically, scoping reviews) this strengthens the aims of a methodology which is based on rigorous and standardized search and synthesis.
4. No exclusion criteria noted.
5. Section for policy and practice implications works well, but would add more areas of further research or gaps as this extensive scoping review provides an important glimpse into this and the authors cited use of Arksey and O'Malley.
6. Further detailed comments below.

Major issues

- Reassess the presentation of search strategy as it is somewhat unclear and difficult to replicate. I would go so far as to say that it is not a standard representation of search strategy and would reformat as boolean string (basically S9 and S17 imply this). If MEDLINE searched via PubMed MeSH headings could be indicated where appropriate, etc.
- How was MEDLINE searched? Via PubMed (I assume)? Via Ovid? Also, was each search ID executed independently, or was it just S18 that yielded initial results? S1 yeilds >6k results alone so former is unlikely. Search fields are specific to which search platform (MH, I believe is CINAHL specific, TX is full text)? Regarding MH, if EMS workers are the focus, why were only EMTs considered as an exact subject heading and not paramedics, firefighters, etc.
- PRISMA-ScR states that at least one database search must be included, although with the limited number of databases searched I highly encourage the rest of the search strategies be presented in an appendix or an additional table. Consider including exact Pubmed/MEDLINE search here and then including all search strings in appendix (consider Bughrara, M. S. et al. Beyond COVID-19: the impact of recent pandemics on medical students and their education: a scoping review. Med. Educ. Online 28, 2139657 (2023).)
- No exclusion criteria noted. For example were study protocols excluded? Non-human studies? Conference abstracts? --> this is notable since you cite PROSPERO as one of the databases you searched
- Please rewrite first paragraph of “Study selection” section as it contrasts with published protocol for unclear reasons. Was a third reviewer not utilized to resolve conflicts as stated in protocol? Also how were studies split up with 7 reviewers? If reviewers in pairs of two that leaves one odd person out. I am left not understanding clearly what you did.
- Discrepancies in number of studies. In one place you say “71 quantatiative” and in another “74 quantitative.” Please fix.
- I consider Delphi methodology to be in the domain of qualitative inquiry although it can have quantitative elements. Please move to qualitative tables
- When comparing tables to “narrative synthesis” sections the counts do not seem to match. Please fix.
- For discussion section one of the stated aims of this scoping review is: “The findings of this scoping review will be used to inform future research to strengthen EMS pandemic preparedness planning.” However no gaps in literature were discussed or areas of future research outside of last sentence (lines 784-786).

Minor issues

- Consider including why librarian or expert search specialist was not consulted to refine search strategy as this would have made for a stronger search methodology.
- PubMed and MEDLINE listed as separate databases. If I’m inferring correctly authors mean that PubMed was used to search MEDLINE so should be cited as PubMed/MEDLINE.

- “Inclusion criteria” section is a bit confusing as presented as a summary introduction and then “more specifically.” Would be more useful and reader friendly to just write out in paragraph form using same “specifically” headings; or get rid of intro sentence and just make sure “specifically” section is complete.
- Mixed methods inclusion criteria is a bit muddled although it is clear what you did in results.
- “epidemics or pandemics” is not well defined narratively. Are non-epidemic outbreaks considered? We know they are from search criteria but text should support this as well.
- Consider changing the words “All international” to global. International typically refers to involving two or more nations (as in international relations) whereas global denotes a global context or scope without implying interactions between countries.
- How did you choose these 6 databases? CINAHL makes sense but why not Web of Science or Scpopus or Embase (as recommended by guidelines for Cochrane), for example?
- Might be helpful to include a table with inception dates of all included databases.
- As MeSH headings pecific only to NLM and clinicaltrials.gov so consider including other terms specific to CINAHL, Cochrane, PsycINFO and PROSPERO if applicable. In search strategy MH and TX somewhat nonspecific.
- Was it noted if missing data were not provided by study authors contacted because not available vs non-response? Did any authors reply to query for missing data?
- Saying studies were “mainly from the USA” is a bit misleading. 32.43% would be a very large minority or the largest proportion. "mainly" implies the majority or >50%. Could include percentages with the remaining study locations you mention as well.
- I prefer one decimal point for averages as two doesn’t really add much meaning for readers.
- What do you mean by “questionnaire study”? Multiple study types use survey instruments and it is not a study methodology. Cross-sectional? Retrospective?
- **Discussion section: w**hat does "prepared" mean? Mentally? with PPE?
- Although not a systematic review it is a bit unclear why EMBASE not searched: https://www.ncbi.nlm.nih.gov/books/NBK209506/table/ttt00116/ 3.1.4 "The three most important sources to search for studies are Cochrane Central Register of Controlled Trials, MEDLINE, and EMBASE."
