# Supplementary material for: Emergency medical service interventions and experiences during pandemics: A scoping review
Source: PLoS One. 2024 Aug 1;19(8):e0304672. doi: 10.1371/journal.pone.0304672 (PMC11293743; doi:10.1371/journal.pone.0304672)
Supplement: Attachment — Submitted filename: emspandemicreview_plosone_responsetoreviewers.docx [file pone.0304672.s004.docx]

| **No** | **Reviewer comments** | **Author response** |
| --- | --- | --- |
| 1 | Reviewer #2: Dear authors,  Although publications on COVID-19 have lost relevance after the wave of articles that "exhausted" and "fatigued" the scientific community, you have managed to present a work that collects evidence in a field little observed and of great relevance for care to emergencies and disasters (such as a pandemic); I congratulate you because the search has been rigorous and extensive, I see that it has faithfully concluded what other authors contributed from their studies and added a judicious and reasonable interpretation of the available evidence. Congratulations. | Thank you for your comments. |
|  | | |
| 2 | Reviewer #1: Please see attached PDF for additional reviewer comments.  Regarding: overall recommendation "minor revision" --> I think this is more along the lines of moderate, or between minor and major  1. Manuscript is technically sound although it does need some review | Thank you - we have responded to all comments and made changes accordingly throughout the manuscript. |
| 3 | 2. No major statistical analysis to speak of in this methodology | Thank you. We have used descriptive statistics, frequency counts and a narrative synthesis, which we felt were more appropriate for a scoping review. |
| 4 | 3. data contained in paper although I would encourage more clear search strategy reporting | We have now included an updated table (Table 1, p. 9), which displays the search strategy (as a Boolean search string) that we used for each database we searched. |
| 5 | 4. Manuscript is overall intelligible although could do with significant review for clarity as many sections somewhat confusing requiring multiple read throughs. | Thank you, we have made extensive changes throughout the manuscript to increase clarity and reduce confusion. We have also revised this paper for clarity with support from our research centre study review group, where colleagues have reviewed the manuscript and helped us make changes accordingly. |
| 6 | Summary of the research  This is an excellent scoping review of EMS interventions and experiences in a pandemic / epidemic / outbreak context with some moderate need for revision. The aim of the authors was to “identify and present the available … evidence of EMS pandemic preparedness, and how this translates into practice.” They hope the findings will inform future research and strengthen policy/practice. The authors searched 6 databases and created a narrative synthesis of all eligible quantitative, qualitative and mixed methods studies. Results were presented in terms of main themes resulting from this review for quantitative and qualitative “arms” of review seperately.  22599 citations were whittled down to 90 pertinent studies.  Quantitative: 7 themes (abstract)  Qualitative: 5 themes (abstract)  Main findings were concern for self and family safety expressed by EMS workers. Although there were concerns for knowledge and training as well and appropriate PPE, EMS personnel were generally “willing and prepared” to report to duty. | Thank you for your comment. |
| 7 | This research fills an important gap in the literature, however one narrative review (using same basic framework of PRISMA-Scr, however much more limited in scope and focused on EMS calls only) is out there:  Alaa Al Amiry & Brian J Maguire (2021) Emergency Medical Services (EMS) Calls During COVID-19: Early Lessons Learned for Systems Planning (A Narrative Review), Open Access Emergency Medicine, 13: 407-414, DOI: 10.2147/OAEM.S324568  Although not critical, consider citing this study in introduction along with [5] and [6]. | Thank you for the suggestion. We have now included this study in our Manuscript (p.5, lines 99-101): “Finally, a narrative review [7] investigated the global increase of EMS calls due to COVID-19 and the reasons behind the bottleneck of EMS calls during the early phase of the pandemic.” |
| 8 | 1. Based on publication criteria this manuscript will meet all criteria with several minor to moderate edits and clarifications. | Thank you - we have responded to all comments and made changes accordingly throughout the Manuscript. |
| 9 | 2. Meets use of PRISMA-Scr checklist as required for best practice in scoping reviews.  Unclear from the manuscript why Arksey and O’Malley was also used although the framework provided therein is rich, detailed and I’m sure was of use to the authors as it is somewhat focused on “the fourth type of scoping study, aimed at identifying gaps in the  existing research literature.” Per my reading this manuscript is a blend of both the 3rd and 4th types of scoping reviews as detailed by Arksey and O’Malley. | Thank you. Indeed, we used the Arksey & O’Malley framework to help us develop and guide the conduct of the review, while at the same time using PRISMA- ScR to ensure the correct and transparent reporting of this review. |
| 10 | 3. Appreciate reflexivity statement, but unclear as to how (for this type of publication specifically, scoping reviews) this strengthens the aims of a methodology which is based on rigorous and standardized search and synthesis. | We agree and have removed this section, thank you. |
| 11 | 4. No exclusion criteria noted. | Thank you, we have now included the following on p.7 (lines 155-159): “Studies were excluded if: they were conducted in the emergency department or hospital; the participants were non-EMS personnel (e.g., hospital nurses, General Practitioners, etc.); the interventions were not implemented during an epidemic or pandemic; and/or the papers were published in a language other than English, due to lack of resources for translation of such papers,” |
| 12 | 5. Section for policy and practice implications works well, but would add more areas of further research or gaps as this extensive scoping review provides an important glimpse into this and the authors cited use of Arksey and O’Malley. | Thank you, we have now included more information on current gaps in literature and possible areas for future research (p. 95, lines 833-844): “In addition, the majority of the reviewed studies were conducted in high-income countries, with predominantly White participants. More studies are, therefore, needed in low- and middle-income countries, with participants from diverse ethnic backgrounds, to evaluate whether the same interventions can be successfully implemented across different countries and populations. Further research with more diverse populations is also needed to explore potential factors affecting EMS providers’ experiences, views, and attitudes towards working during pandemics. Equally, it would be crucial to investigate the long-term impact of working in a pandemic on the well-being and working conditions of Emergency Medical Services and how these may be affected by the trajectory of the disease outbreak. Finally, the majority of the included studies did not report any or had missing data on the age, gender and/or ethnicity of their participants. Better reporting is, therefore, needed of demographic characteristics of the participants in published papers in the future.” |
| 13 | 6. Further detailed comments below. | Thank you, please see below for our detailed responses. |
| 14 | **Major issues**  • Reassess the presentation of search strategy as it is somewhat unclear and difficult to replicate. I would go so far as to say that it is not a standard representation of search strategy and would reformat as boolean string (basically S9 and S17 imply this). If MEDLINE searched via PubMed MeSH headings could be indicated where appropriate, etc. | We have now included an updated table (Table 1, p. 9), which displays the search strategy (as a Boolean search string) that we used for each of the databases we searched |
| 15 | • How was MEDLINE searched? Via PubMed (I assume)? Via Ovid? Also, was each search ID executed independently, or was it just S18 that yielded initial results? S1 yeilds >6k results alone so former is unlikely. Search fields are specific to which search platform (MH, I believe is CINAHL specific, TX is full text)? Regarding MH, if EMS workers are the focus, why were only EMTs considered as an exact subject heading and not paramedics, firefighters, etc. | We searched these databases separately. We accessed MEDLINE via EBSCOhost. We then accessed PubMed and ran the searches in PubMed as well. In hindsight, we recognise that we could have just searched only one of these databases.  We did run each search separately, but we understand that Table 1 might be a bit confusing (including the abbreviations for each search field), so we have taken your advice and created a new Table 1 (see answer above to Reviewer comments 4 & 16 as well).  We were only interested in ambulance personnel and no other emergency services, such as firefighters, etc. On reflection, we could have included EMS as a subject heading; however, it was felt that including variations of EMS and paramedic terms in full text fields alongside our supplementary searches (references/citation searches) would result in a reasonably comprehensive search to identify the relevant literature. |
| 16 | • PRISMA-ScR states that at least one database search must be included, although with the limited number of databases searched I highly encourage the rest of the search strategies be presented in an appendix or an additional table. Consider including exact Pubmed/MEDLINE search here and then including all search strings in appendix (consider Bughrara, M. S. et al. Beyond COVID-19: the impact of recent pandemics on medical students and their education: a scoping review. Med. Educ. Online 28, 2139657 (2023).) | We have now included an updated table (Table 1, p. 9), which displays the search strategy (as a Boolean search string) that we used for each database we searched |
| 17 | • No exclusion criteria noted. For example were study protocols excluded? Non-human studies? Conference abstracts? --> this is notable since you cite PROSPERO as one of the databases you searched | Thank you, we have now included the following on p.7 (lines 155-159): “Studies were excluded if: they were conducted in the emergency department or hospital; the participants were non-EMS personnel (e.g., hospital nurses, General Practitioners, etc.); the interventions were not implemented during an epidemic or pandemic; and/or the papers were published in a language other than English, due to lack of resources for translation of such papers.”.  Regarding PROSPERO, we did indeed look for existing protocols and have added this information under “Information sources and search strategy”, p.7 (lines 166-167): “PROSPERO was also searched for protocols of existing (completed or ongoing) systematic reviews.” |
| 18 | • Please rewrite first paragraph of “Study selection” section as it contrasts with published protocol for unclear reasons. Was a third reviewer not utilized to resolve conflicts as stated in protocol? Also how were studies split up with 7 reviewers? If reviewers in pairs of two that leaves one odd person out. I am left not understanding clearly what you did. | Thank you, we meant that eligibility was discussed among the whole team of reviewers and not just with a third reviewer. There was also one reviewer (DL) that formed part of more than one pair of reviewers assessing eligibility of references.  We have now corrected the above in p. 9-10 of the Manuscript (lines 199-200 & 204) |
| 19 | • Discrepancies in number of studies. In one place you say “71 quantitative” and in another “74 quantitative.” Please fix. | Thank you, we have made changes accordingly throughout the paper.  There are 90 studies altogether: 73 quantitative (including the quantitative arms of 2 mixed methods studies) & 18 qualitative studies (including the qualitative arms of 3 mixed methods studies, 2 surveys analysed qualitatively & 1 text/opinion study). Also, we have provided more details in p.11-12 to show what these numbers mean. |
| 20 | • I consider Delphi methodology to be in the domain of qualitative inquiry although it can have quantitative elements. Please move to qualitative tables | Thank you for your comment. We have checked this paper by Belfroid and colleagues (Belfroid E, Timen A, van Steenbergen JE, Huis A, Hulscher ME. Which recommendations are considered essential for outbreak preparedness by first responders? BMC Infect Dis. 2017; 17:195. <https://doi.org/10.1186/s12879-017-2293-0>) and they’ve used a quantitative design and only presented quantitative findings (i.e., the results from the questionnaires that were used to gather the participants’ recommendations) and no qualitative findings. Therefore, we feel it is more appropriate to categorise the approach used as a quantitative methodology. |
| 21 | • When comparing tables to “narrative synthesis” sections the counts do not seem to match. Please fix. | Thank you- we have removed 1 reference [35] from the Theme “Call volumes, ambulance response times and triage (logistics of service delivery)” (p.70) and another one [73] from the Theme “Identifying patients, testing & vaccinations” (p.61-62).  We have also made corrections accordingly, throughout the manuscript, where percentages of studies are reported |
| 22 | • For discussion section one of the stated aims of this scoping review is: “The findings of this scoping review will be used to inform future research to strengthen EMS pandemic preparedness planning.” However no gaps in literature were discussed or areas of future research outside of last sentence (lines 784-786). | Thank you, we have now expanded this section and reported on both current gaps in literature and possible areas for future research (p. 95, lines 829-844): “In addition, the majority of the reviewed studies were conducted in high-income countries, with predominantly White participants. More studies are, therefore, needed in low- and middle-income countries, with participants from diverse ethnic backgrounds, to evaluate whether the same interventions can be successfully implemented across different countries and populations. Further research with more diverse populations is also needed to explore potential factors affecting EMS providers’ experiences, views, and attitudes towards working during pandemics. Equally, it would be crucial to investigate the long-term impact of working in a pandemic on the well-being and working conditions of emergency medical services and how these may be affected by the trajectory of the disease outbreak. Finally, the majority of the included studies did not report any or had missing data on the age, gender and/or ethnicity of their participants. Better reporting is, therefore, needed of demographic characteristics of the participants in published papers in the future.” |
| 23 | **Minor issues**  • Consider including why librarian or expert search specialist was not consulted to refine search strategy as this would have made for a stronger search methodology. | Thank you, we have now included this as a limitation of our review (p. 96, lines 863-867): “Another limitation of this review was the absence of a subject librarian review of the search strategy, as this would have made for a stronger methodology. That said, many search terms were searched for in full text fields and there were further supplementary searches conducted to identify any additional references, which produced a high number of references to be screened”. |
| 24 | • PubMed and MEDLINE listed as separate databases. If I’m inferring correctly authors mean that PubMed was used to search MEDLINE so should be cited as PubMed/MEDLINE. | We searched these databases separately. We accessed MEDLINE via EBSCOhost. We then accessed PubMed and ran the searches in PubMed as well. In hindsight, we recognise that we should have just searched only one of these databases. |
| 25 | • “Inclusion criteria” section is a bit confusing as presented as a summary introduction and then “more specifically.” Would be more useful and reader friendly to just write out in paragraph form using same “specifically” headings; or get rid of intro sentence and just make sure “specifically” section is complete. | Thank you, we have made changes accordingly in p. 6-7 |
| 26 | • Mixed methods inclusion criteria is a bit muddled although it is clear what you did in results. | We have re-worded the mixed-methods inclusion criteria in p. 7 (lines 153-154) to make it clearer: “Data from multi-methods studies were extracted into the respective quantitative and/or qualitative arm of this review and synthesised accordingly.”  We have also re-worded parts of the results section (p.11-12) to make this clearer. |
| 27 | • “epidemics or pandemics” is not well defined narratively. Are non-epidemic outbreaks considered? We know they are from search criteria but text should support this as well. | Thank you for your comment. We were only interested in epidemic/pandemic outbreaks. We included broad search terms in our strategy to be as inclusive as possible, when identifying relevant references and not miss any potential relevant ones. When conducting the full-text screening of such references, though, we excluded any references that discussed non-epidemic/pandemic disease outbreaks.  We have also included the following in our exclusion criteria to make this clearer (see response to Reviewer comments 11 & 17 as well): “Studies were excluded if: they were conducted in the emergency department or hospital; the participants were non-EMS personnel (e.g., hospital nurses, General Practitioners, etc.); **the interventions were not implemented during an epidemic or pandemic**; and/or the papers were published in a language other than English, due to lack of resources for translation of such papers.” |
| 28 | • Consider changing the words “All international” to global. International typically refers to involving two or more nations (as in international relations) whereas global denotes a global context or scope without implying interactions between countries. | Thank you, we have made changes accordingly on p.6 (where we are describing findings of a study where the authors use the word “international” we have left it as is) |
| 29 | • How did you choose these 6 databases? CINAHL makes sense but why not Web of Science or Scpopus or Embase (as recommended by guidelines for Cochrane), for example? | Thank you, we did use Web of Science as well but failed to mention this in the Methods. We have now added this information in the Methods section (p. 7, lines 163-164) & have included the full search strategy for Web of Science in Table 1 as well. |
| 30 | • Might be helpful to include a table with inception dates of all included databases. | Thank you, we have now included this information in Table 1 (p. 9). |
| 31 | • As MeSH headings pecific only to NLM and clinicaltrials.gov so consider including other terms specific to CINAHL, Cochrane, PsycINFO and PROSPERO if applicable. In search strategy MH and TX somewhat nonspecific. | Thank you, we have updated Table 1 (p. 9), which now includes full search strategies for each database. |
| 32 | • Was it noted if missing data were not provided by study authors contacted because not available vs non-response? Did any authors reply to query for missing data? | We have looked through our records (both emails and computer files) and we have not found any relevant correspondence. We, therefore, have removed this from our Manuscript (p. 10, lines 212-213). |
| 33 | • Saying studies were “mainly from the USA” is a bit misleading. 32.43% would be a very large minority or the largest proportion. "mainly" implies the majority or >50%. Could include percentages with the remaining study locations you mention as well. | We have now corrected this and have also included percentages for all study locations (p.12, lines 274-277): “The 73 quantitative studies (including the quantitative arms of two mixed-methods studies) (Tables 2 – 8) were published between 2004 and 2022 and were from the USA (n= 24; 32.9%) or Canada (n= 2; 2.7%), Europe (n= 21; 28.8%), Asia (n= 22; 30.1%), and Australia (n= 3; 4.1%), while one Delphi study [17] included an international panel of experts (1.4%). ”  We have made a similar change for the study characteristics of the qualitative synthesis (p. 73, lines 534-535): “….were predominantly from the USA (33.3%).” |
| 34 | • I prefer one decimal point for averages as two doesn’t really add much meaning for readers. | Thank you, we have made changes accordingly throughout the paper |
| 35 | • What do you mean by “questionnaire study”? Multiple study types use survey instruments and it is not a study methodology. Cross-sectional? Retrospective? | Thank you, we have corrected this throughout the manuscript to now read “cross-sectional, questionnaire studies”. The only exceptions were Lee et al. (2022) [64], which was a prospective questionnaire study (p.44), and Pozner et al. 2022) [86], a retrospective questionnaire study (p. 60).  In addition, under the “Study characteristics” of the quantitative studies, we have made a correction and the text is now “A large proportion of studies (n= 15; 20.5%) were cross-sectional, questionnaire studies.” (p.65, lines 323-324) |
| 36 | • **Discussion section**: what does "prepared" mean? Mentally? with PPE? | Thank you - we have clarified this: “This scoping review explored the available quantitative and qualitative evidence of EMS pandemic preparedness (i.e., to be able to respond and take action effectively on a personal and organisational level), and how this translates into practice.” (p. 91, lines 741-742) |
| 37 | • Although not a systematic review it is a bit unclear why EMBASE not searched:  https://www.ncbi.nlm.nih.gov/books/NBK209506/table/ttt00116/ 3.1.4 "The three most important sources to search for studies are Cochrane Central Register of Controlled Trials, MEDLINE, and EMBASE." | Thank you. We chose instead to search Web of Science Core Collection, which was not stated in our original submission. We have now included this in the Methods section (p. 7, lines 163-164) & have included the full search strategy for Web of Science in Table 1 as well. |
